# Flexible High Lithium‐Ion Conducting PEO‐Based Solid Polymer Electrolyte with Liquid Plasticizers for High Performance Solid‐State Lithium Batteries

**DOI:** 10.1002/open.202400041

**Published:** 2024-04-15

**Authors:** Ayaka Abe, Daisuke Mori, Zhichao Wang, Sou Taminato, Yasuo Takeda, Osamu Yamamoto, Nobuyuki Imanishi

**Affiliations:** ^1^ Graduate School of Engineering Mie University Tsu Mie 514-8507 Japan; ^2^ Research Center for Integrated Materials and Interfaces for Sustainable Energy Mie University Tsu Mie 514-8507 Japan

**Keywords:** composite, liquid plasticizer, lithium anode, polymer electrolyte, solid lithium-ion conductor

## Abstract

Lithium‐ion secondary batteries (LIB) with high energy density have attracted much attention for electric vehicle (EV) applications. However, LIBs have a safety problem because these batteries contain a flammable organic electrolyte. As such, all‐solid secondary batteries that are not flammable have been extensively reported recently. In this study, we have focused on polymer electrolytes, which is flexible and is expected to address the safety problem. However, the conventional polymer electrolytes have low electrial conductivity at room temperature. Various attempts have been made to solve this problem, such as the addition of inorganic fillers and ionic liquids; however, these composite polymer electrolytes have not yet reached a practical level of lithium‐ion conductivity. In this study, high electrical conductivity and lithium dendrite formation‐free PEO based composite electrolytes are developed with both a filler of Li_6,4_La_3_Zr_1.4_Ta_0.6_O_12_ and liquid plasticizers of tetraethylene glycol dimethyl ether and 1,2 dimethoxyethane. The proposed flexible polymer electrolyte shows a high electrical conduciviy of 6.01×10^−4^ S cm^−1^ at 25 °C.

## Graphical abstract

High Li‐ion conductivity flexible composite polymer electrolytes based on polyethylene oxide(PEO_18_LiTFSI) with the addition of a solid Li^+^ ion conductor of Li_6.4_La_3_Zr_1.4_Ta_0.6_O_12_ and liquid plasticizers of tetraethylene glycol dimethyl ether and 1,2 dimethoxyethane are developed. The proposed composite electrolyte shows a highest electrical conductivity of 6.01×10^−4^ S cm^−1^ at 25 °C

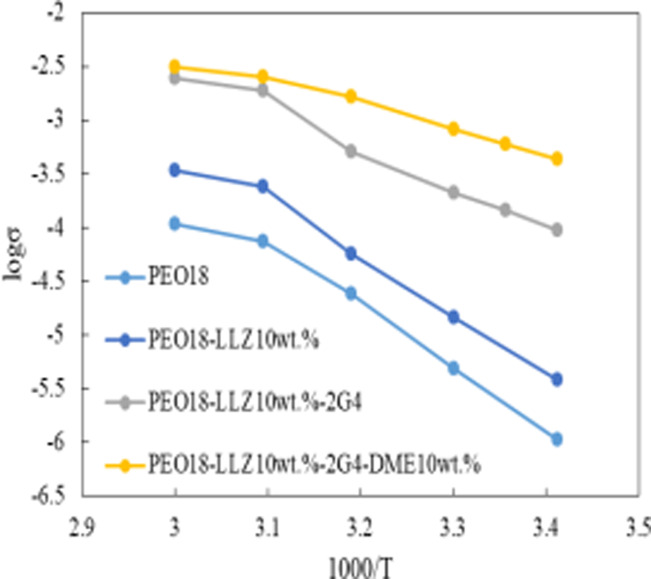

## Introduction

More than half of new passenger vehicles with an internal combustion engine (ICE) will be converted to electrical vehicles (EVs) with batteries or fuel cells by 2035 with the aim to reduce carbon dioxide emissions.^[1]^ Vehicles with batteries or fuel cells that have comparable performance and prices to those of ICEs have not yet been developed. The major technical hurdle for EVs is the insufficient storage capacity of the present batteries, which severely limits the practical range of EVs. The usable energy density of gasoline in automotive applications is estimated to be approximately 1700 Wh kg^−1^,^[2]^ which is approximately 10‐fold that of the current energy density of lithium‐ion secondary batteries (LIBs), which are typically between 100 and 200 Wh kg^−1^ (cell level). However, the EV market demands an even higher specific energy of more than 500 Wh at the cell level, and a low cost of below US$ 100 kWh^−1^ at the package level.^[3]^ At present, next generation high energy density batteries such as lithium‐air,^[4]^ lithium‐sulfur,^[4]^ and high‐energy lithium metal batteries^[5]^ have been extensively studied. All‐solid‐state batteries have also been undergone widespread study due to their inherently high safety characteristics. Safety for high capacity batteries is the most important requirement for EVs. The lithium metal batteries and next generation lithium‐air and lithium‐sulfur batteries contain much flammable liquid electrolyte and active lithium metal. Two types of all‐solid‐state batteries have been developed, polymer and inorganic sold electrolyte batteries. Kanno and co‐workers^[6]^ reported a high performance all‐solid‐state lithium battery with a sulfide based solid electrolyte with extremely high lithium‐ion conductivity of 2.5×10^−2^ S cm^−1^ at room temperature. Now many groups around the world have developed batteries with this electrolyte for EV applications. However, this electrolyte is quite hydroscopic and the reaction product with water is poisonous H_2_S. The weight loss for protection from damage by an accident should be accounted for in the cell energy density.

We have a long history of work on lithium‐ion conducting polymer electrolyte batteries from the first report in 1973 by Fenton et al.^[7]^ Lithium polymer electrolyte batteries were reported by Armand et al. in 1978.^[8]^ In the early 1990s, 3 M and Hydro‐Quebec developed lithium polymer electrolyte batteries for EVs.^[9]^ In the first stage, the lithium‐ion conductivity at room temperature was as low as 10^−6^ S cm^−1^ and a high lithium‐ion conductivity of more than 10^−4^ S cm^−1^ was achieved after melting of the polymer at more than 60 °C; therefore, lithium polymer batteries have been operated at more than 60 °C. The room temperature lithium‐ion conductivity of polymer electrolytes has been significantly improved by the addition of inorganic oxide fillers^[10]^ and less flammable ionic liquids.^[11]^ The conductivity of polyethylene oxide (PEO)‐lithium bis(trifluoromethanesulfonyl)imide (LiTFSI) (PEO_10_LiTFSI) at 30 °C (3.5×10^−6^ S cm^−1^) was enhanced to 1.6×10^−4^ S cm^−1^ by the addition of 20 vol % Li_6,4_La_3_Zr_1.4_Ta_0.6_O_12_ (LLZTO) with a particle size of 200 nm.^[12]^ LLZTO is known as a high lithium‐ion conducting solid electrolyte with a room temperature conductivity of approximately 7×10^−4^ S cm^−1^. A composite of PEO_18_LiTFSI and an ionic liquid of tetraethylene glycol dimethyl ether (G4) showed a conductivity of 6.8×10^−5^ S cm^−1^ at 25 °C.^[13]^

The other important requirement of a polymer electrolyte for lithium batteries is reversibility for lithium deposition and stripping on the lithium metal electrode. The lithium metal anode is the best candidate for high energy density rechargeable batteries due to its high specific capacity of 3,860 mA g^−1^ and a low standard electrochemical redox potential (−3.04 V vs. SHE).^[14]^ However, lithium dendrite formation during the lithium deposition process is a critical issue for safe rechargeable batteries because lithium dendrites can penetrate the separator and reach the cathode, which causes an electrical short circuit of the cell.^[15]^ A Li/1 M LiPF_6_ in ethylene carbonate (EC)‐dimethyl carbonate (DMC)‐ethyl methyl carbonate (EMC) (1 : 1 : 1, v/v)/Li with a 1 mm thick Teflon separator short‐circuited after 11 min polarization at 1.0 mA cm^−2^.^[16]^ A fine pore size polyethylene separator has been used to suppress lithium dendrite formation. Choudhury and Archer^[17]^ reported the cycle performance with a conventional electrolyte of 1 M LiPF_6_ in EC‐DMC with a Celgard 3510 separator. A short circuit was observed after around 870 h at 1 mA cm^−2^ with 4 h cycles. The time until short‐circuit was decreased with an increase of the current density; 336 h at 2 mA cm^−2^ for 2 h cycles and 80 h at 4 mA cm^−2^ for 1 h cycles. An electrolyte of 4 M LiLiFSI in 1,2 dimethoxyethane (DME) with the Celgard separator showed no short‐circuit due to dendrite formation for 1000 cycles at 1 mA cm^−1^ with 1 h cycles at room temperature.^[18]^ However, the electrolyte is not acceptable for the large size batteries required for EVs, because of the flammability, high cost and high viscosity of the electrolyte. The onset of lithium dendrite formation with a typical polymer electrolyte of PEO_18_LiTFSI occurs after 15 h polarization at 0.5 mA cm^−2^ and 60 °C.^[19]^ The lithium electrode capacity until the onset of lithium dendrite formation with a 10 μm copper substrate is 688 mAh g^−1^. The lithium dendrite formation onset time was delayed by the addition of an ionic liquid of N‐methyl‐N‐propylpiperidinium bis(trifluoromethanesulfonyl)imide (PP13TFSI) and nano‐silica.^[19]^ The lithium dendrite formation onset time for polyethylene PEO_18_LiTFSI‐1.44 PP13TFSI‐10 wt % SiO_2_ was reported to be 35 h at 0.5 mA cm^−2^ and 60 °C. The capacity of the lithium electrode including a 10 μm copper substrate was 2154 mAh g^−1^, which is more than 5 times higher than that of a carbon electrode.

In this study, we have focused on room temperature operation for the electrolyte of polymer lithium batteries. A composite PEO based polymer electrolyte with a lithium‐ion conducting solid oxide electrolyte filler of LLZTO and G4 as a plasticizer has been proposed, and the addition of DME to the composite electrolyte has been examined with an aim to improve the lithium electrode performance.

## Results and Discussion

**Figure 1:**
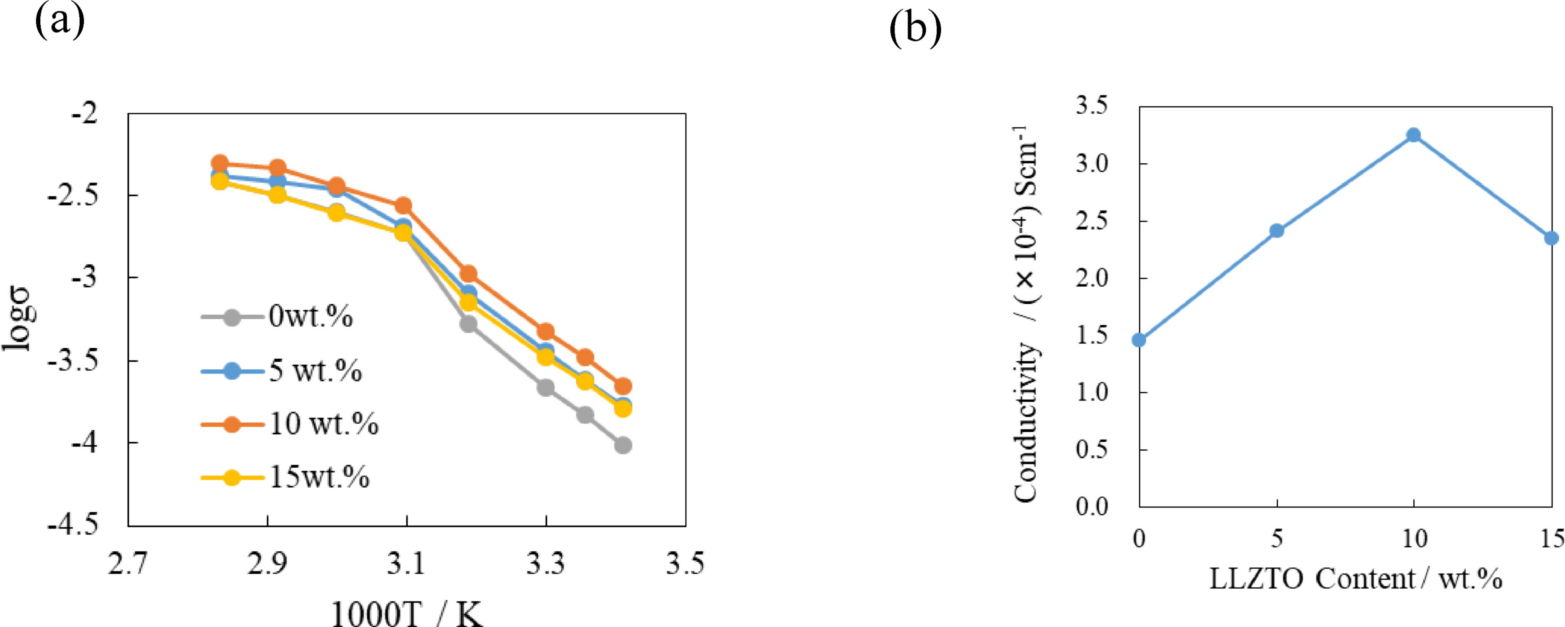
(a) Temperature dependence of electrical conductivity and (b) composition dependence of electrical conductivity at 25 °C for PEO_18_LiTFSI‐2G4‐x LLZTO.

We have previously reported^[13]^ that the electrical conductivity of a typical PEO based lithium ion‐conductor of PEO_18_LiTFSI was as low as 5.64×10^−6^ S cm^−1^ at 25 °C and the lithium‐ion transport number was 0.24. The electrical conductivity and the lithium‐ion transport number were enhanced to 6.08×10^−5^ S cm^−1^ and 0.54, respectively, by the addition of 2 moles of G4 to PEO_18_LiTFSI (34 wt %). There have been many reports on the lithium‐ion conductivity enhancements of polymer electrolytes by the addition of ion conducting oxide fillers.[^12^, ^20^, ^21^, ^22^] Enhancement of the electrical conductivity of a polymer electrolyte with a low content of solid oxide fillers (ceramic in polymer) was explained by a decrease of the crystallinity of the PEO matrix and/or interface conductivity of the oxide fillers.[^21^, ^22^] In this study, we have studied the synergistic effect of the addition of LLZTO and G4 into PEO_18_LiTFSI. Figure 1 shows the temperature dependence of the electrical conductivity and the compositional dependence of the electrical conductivity at 25 °C for PEO_18_LiTFSI‐2G4‐x wt % LLZTO.

The electrical conductivity of PEO_18_LiTFSI‐2G was increased by the addition of LLZTO up to 10 wt % LLZTO, after which it decreased. This compositional dependence of the electrical conductivity of PEO_18_LiTFSI‐2G4‐xLLZTO is similar to that of PEO_8_TFSI‐xLLZTO.^[23]^ The highest electrical conductivity of 3.25×10^−4^ S cm^−1^ at 25 °C for PEO_18_LiTFSI‐2G4‐10 wt % LLZTO is higher than that of PEO_8_TFSI‐10 wt % LLZTO at 30 °C (1.17×10^−4^ S cm^−1^). The increase of the electrical conductivity was explained by the increase in the free volume for the motion of the PEO chain segment, which may be deceased by more than that for the 10 wt % addition of LLZTO.^[23]^ Figure S1 and S2 show XRD patterns and Raman spectra of PEO_18_LiTFSI‐2G4‐xLLZTO, respectively. No significant difference between PEO_18_LiTFSI‐2G4 with and without LLZTO was observed in the XRD patterns and Ramn spectra; however, the intensity of the XRD diffraction lines of PEO_18_LiTFSI‐2G4‐x LLZTO were decreased with an increase in x.

**Figure 2:**
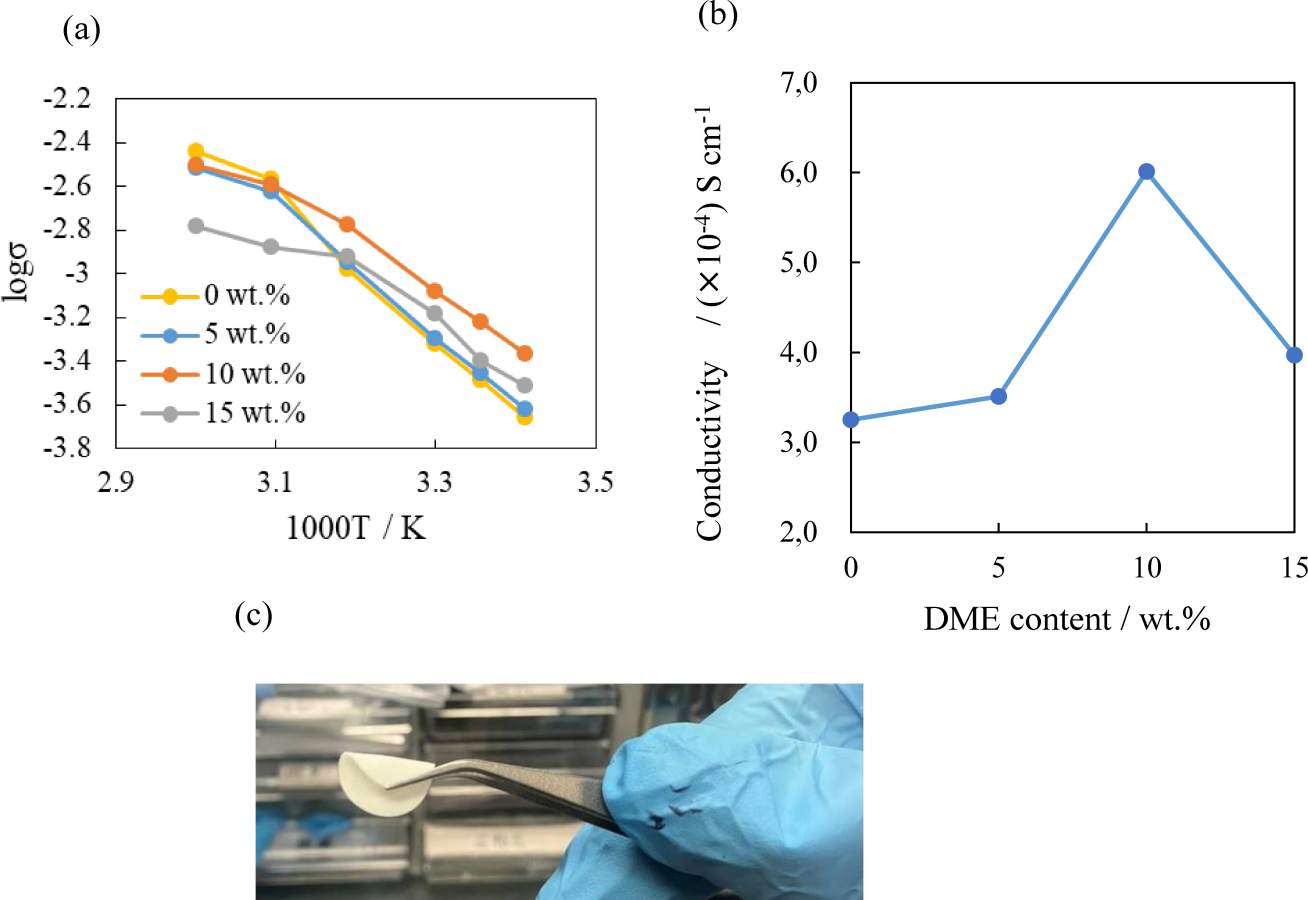
(a) Temperature dependence of the electrical conductivity, (b) compositional dependence of the electrical conductivity at 25 °C for PEO_18_LiTFSI‐2G4‐10 wt % LLZTO‐xDME, and (c) photograph of PEO_18_LiTFSI‐2G‐10 wt % LLZTP‐15 wt % DME electrolyte.

Luu et al.^[24]^ reported a high electrical conductivity for the PEO_18_TFSI‐ Li_7_La_3_Zr_1.76_Al_0.24_O_12_ composite electrolyte with the addition of a liquid plasticizer of succinonitrile (SN). The composite electrolyte of PEO_18_LiTFSI‐7.5 wt % LLZAlO‐15 wt % SN showed a high electrical conductivity of 4.17×10^−4^ S cm^−1^ at room temperature. More recently, Zhang et al.^[25]^ also reported a higher ionic conductivity of 7.16×10^−4^ S cm^−1^ for PEO_18_LiClO_4_‐15 wt % LLZTO‐60 wt % SN. The effect of the addition of a small amount of a low molecular weight solvent of DME into PEO_18_LiTFSI‐2G4‐10 wt % LLZTO has been examined with an aim to enhance the electrical conductivity of the PEO based composite electrolyte. Figure 2 shows the temperature dependence of the electrical conductivity and the compositional dependence of the electrical conductivity at 25 °C for PEO_18_LiTFSI‐2G4‐10 wt % LLZTO‐xDME. The compositional dependence curve of the electrical conductivity for PEO_18_LiTFSI‐2G4‐10 wt % LLZTO‐xDME at 25 °C shows a maximum of 6.01×10^−4^ S cm^−1^ at 10 wt % DME. The conductivity of this composite electrolyte is higher than those of previously reported flexible composite polymer electrolytes,[^26^, ^27^] except for that with a high content of 60 wt % SN..^[25]^ A 0.1 mm thick self‐supporting film of the composite polymer electrolyte with 10 wt % DME was easily prepared by a casting method. The specific area resistance of 16 Ω cm^2^ is acceptable for the electrolyte in practical batteries if the electrode polarization is not too high. Figure 2(c) shows a photograph of the flexible composite electrolyte of PEO_18_LiTFSI‐2G4‐10 wt % LLZTO‐15 wt % DME. The stress vs strain curves of the composite electrolyte studied are schown in Figure S3. A similar compositional dependence of the electrical conductivity was also observed for PEO_18_LiTFSI‐10 wt % LLZTO‐x DME at 25 °C. The highest conductivity of 2.25×10^−4^ S cm^−1^ was observed for the addition of 10 wt % DME as shown in Figure S4. This result suggests that diffusion of lithium‐ions in the plasticizer phase of G4‐DME in the PEO matrix is disturbed by an increased solvation of lithium ions with a high content DME.

**Figure 3:**
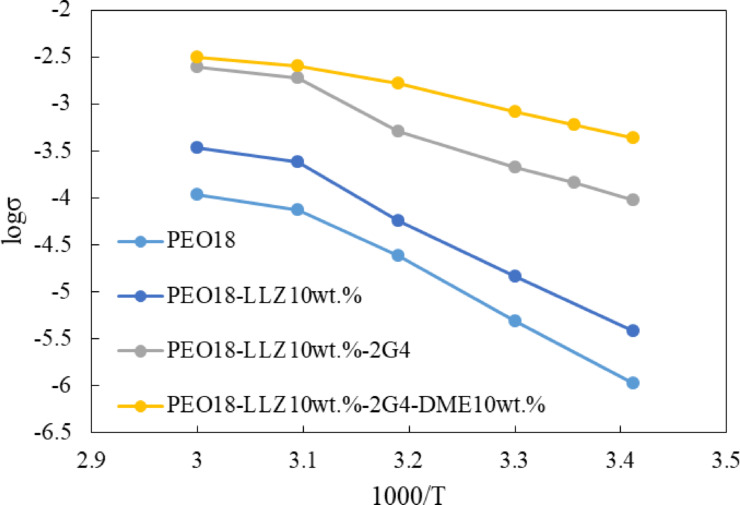
Temperature dependence of the electrical conductivity of PEO based composite electrolytes.

The temperature dependence of the PEO based composite electrolytes and the activation energy for conduction are summarized in Figure 3 and Table 1.The activation energies for electrical conduction were estimated at near to room temperature. The activation energy of 47.5 kJ mol^−1^ for the conduction of PEO_18_‐2G4‐10 wt % LLZTO‐10 wt % DME is considerably lower than those of 117 kJ mol^−1^ for PEO_18_LiTFSI and 78.2 kJ mol^−1^ for PEO_18_LiTFSI‐2G4.^[13]^ The composite electrolyte of PEO_10_LiTFSI‐10 vol % LLZTO also showed a lower activation energy of 54 kJ mol^−1^.^[28]^ The activation energy of the composite electrolyte with DME and LLZTO is comparable to those of high lithium‐ion conducting solid electrolytes such as Li_7_La_3_Zr_2_O_12_ and Li_1.2_Al_0.2_Ti_1.8_(PO_4_)_3_.^[29]^ The low activation energy for the conduction of the composite electrolyte with DME suggests that the lithium‐conduction path in the composite with DME is different from that in the composite without DME. The low activation energy suggests that lithium‐ions may be transported through a liquid‐like phase in the PEO matrix.

**Table 1:** Activation energies for conduction in PEO based electrolytes.

Electrolyte	Conductivity at 25 °C	Activation energy (kJ mol^−1^)	Ref.
PEO_18_LiTFSI‐2G4‐	6.01×10^−4^	47.5	This study
10 wt % LLZTO‐10 wt % DME			
PEO_18_LiTFSI‐2G4‐	3.25×10^−4^	64.5	This study
10 wt % LLZTO			
PEO_18_LiTFSI‐10 wt % LLZTO	7.5×10^−5^	95.6	This study
PEO_18_LiTFSI	3×10^−6^	117	This study
PEO_18_LiTFSI	5.64×10^−6^	115.7	13
PEO_18_LiTFSI‐2G4	6.08×10^−5^	78.2	13
PEO_10_LiTFSI‐20 vol % LLZTO	1.6×10^−4^	54	28
PEO_8_LiTFSI‐10 wt % LLZTO	1.17×10^−4^	51	23

**Figure 4:**
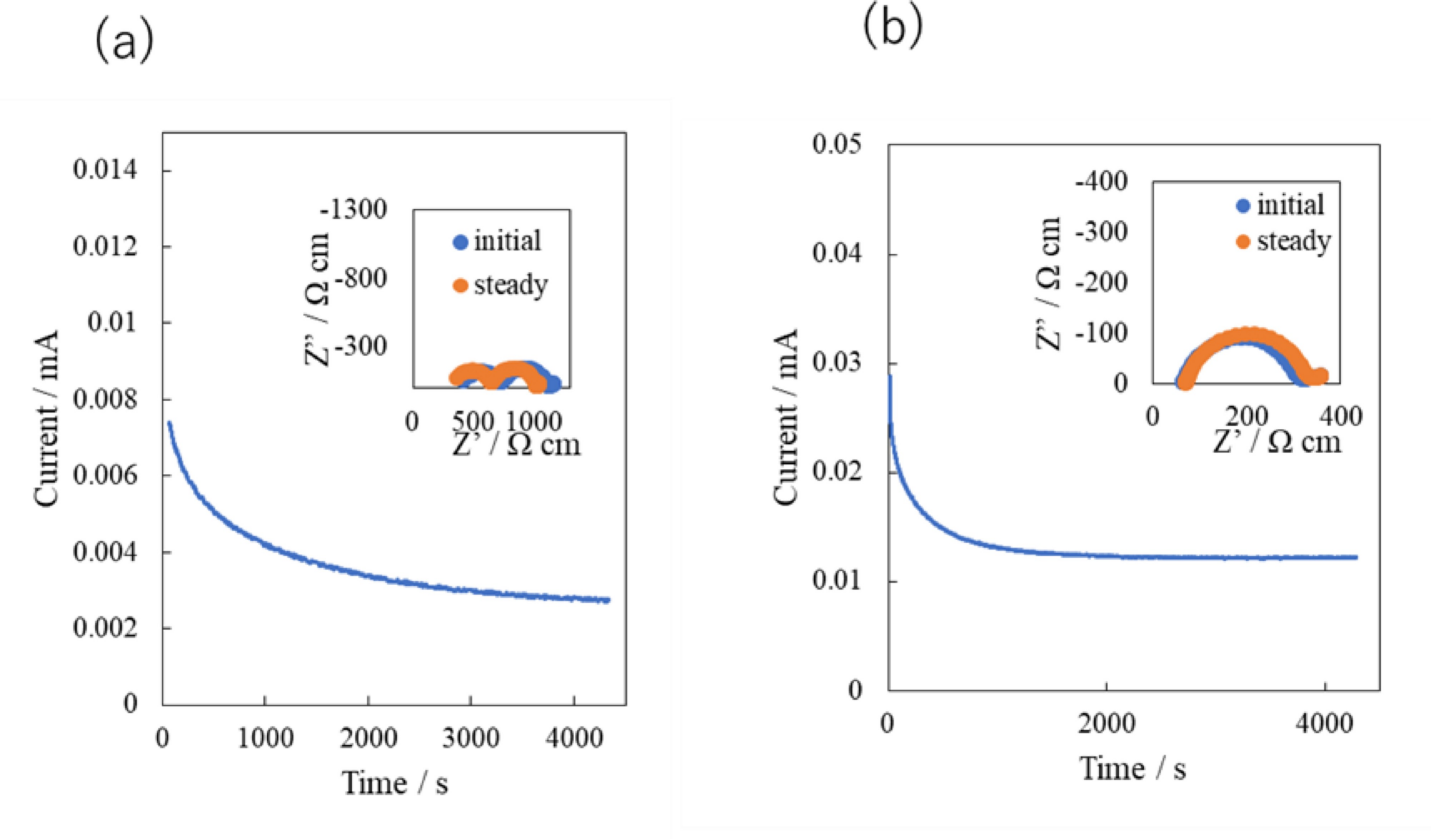
Current decays with time upon application of a 10 mV DC bias and corresponding impedance profiles of the cells befor and after polarization for the (a) Li/PEO_18_LiTFSI‐2G4‐10 wt % LLZTO/Li cell and (b) Li/PEO_18_LiTFSI‐2G4‐10 wt % LLZTO‐10 wt % DME/Li.

Lithium‐ion transport numbers were measured using the method reported by Evans et al.^[30]^ Figure 4 shows the current decay curve upon application of a 10 mV DC bias and the impedance profile changes before and after polarization for the Li/PEO_18_LiTFSI‐2G4‐10 wt % LLZTO/Li and Li/PEO_18_LiTFSI‐2G4‐10 wt % LLZTO‐10 wt % DME/Li cells at 25 °C. The lithium‐ion transport numbers (t_Li+_) were calculated to be 0.20 for the comnposite electrolyte without DME and 0.12 with DME, which is lower than that of PEO_18_LiTFSI (0.24) and lower than that PEO_18_LiTFSI‐2G4 (0.54).^[13]^

**Figure 5:**
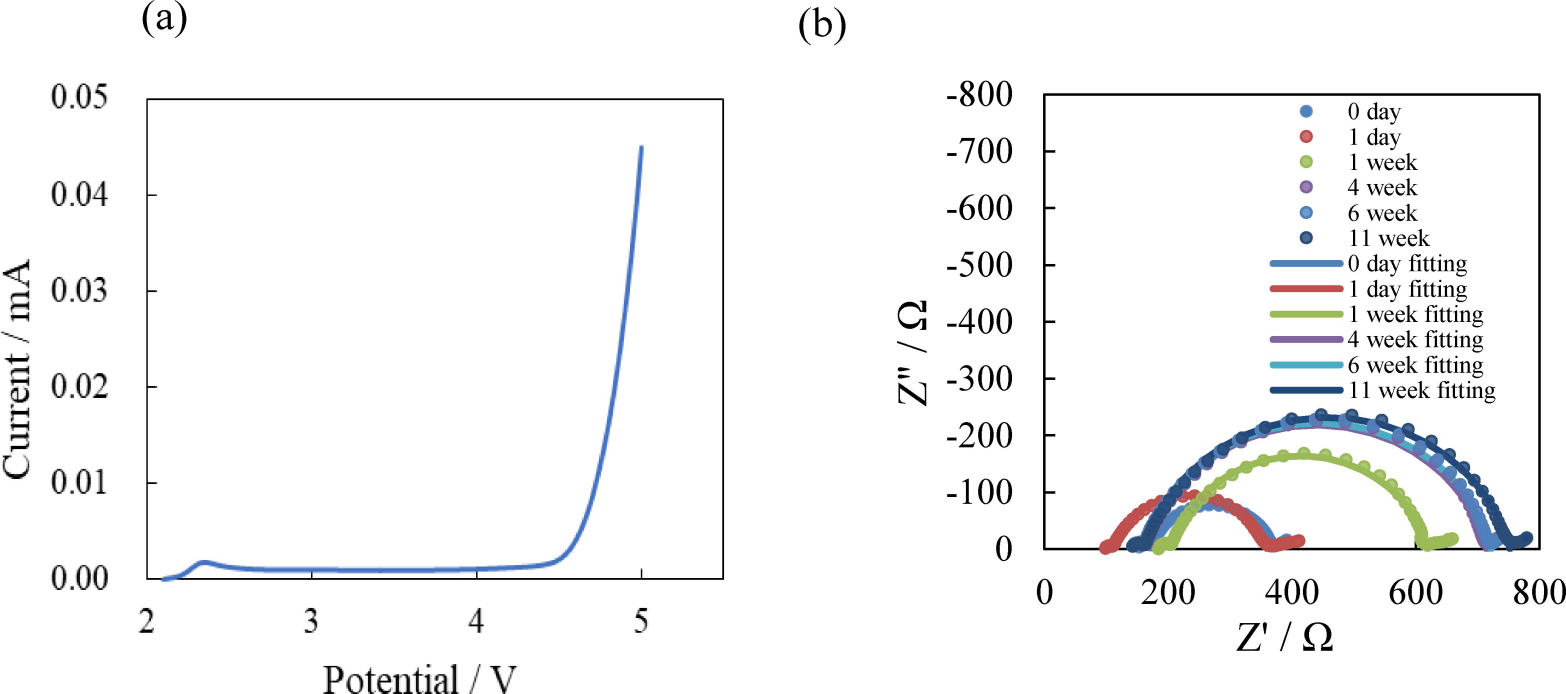
(a) Linear sweep voltammogram for the Li/PEO_18_LiTFSI‐2G4‐10 wt % LLZTO‐10 wt % DME/SS cell with a scan rate of 1 mV s^−1^. (b) Change in the impedance profile with the storage time at 25 °C for the Li/PEO_18_LiTFSI‐2G4‐10 wt % LLZTO‐10 wt % DME/Li cell.

The stable electrochemical potential window of PEO_8_LiTFSI was recorded to be 4.3 V vs. Li/Li^+^.^[23]^ Figure 5(a) shows a linear sweep voltammogram for the Li/PEO_18_LiTFSI‐2G4‐10 wt % LLZTO‐10 wt % DME/stainless steel (SS) cell. The composite electrolyte containing LLZTO, G4 and DME is stable up to 4.4 V. The stability of the interface between the composite electrolyte and lithium is critical factor for the lithium metal batteries with the polymer electrolyte. The high interface resistance of around 400 Ω at 55 °C was reported for the Li/PEO_8_LiTFSI‐10 wt % LLZTO/Li cell.^[23]^ Figure 5(b) shows the change in the impedance for the Li/PEO_18_LiTFSI‐2G4‐10 wt %LLZTO‐10 wt % DME/Li cell with the storage time at 25 °C. The impedance profiles shows two semicircles; the small semicircle in the high frequency range corresponds to the grain boundary resistance of the composite electrolyte and that in the low frequency range to the charge transfer resistance and the interface resistance between the composite electrolyte and lithium electrode.^[31]^ The size of the semicircle in the low frequency was increased with the storage period, which may be due to the formation of a high resistance layer on the lithium electrode. No significant increase of the interface resistance was observed after one week. The steady cell impedance was around 750 Ω at 25 °C.

**Figure 6:**
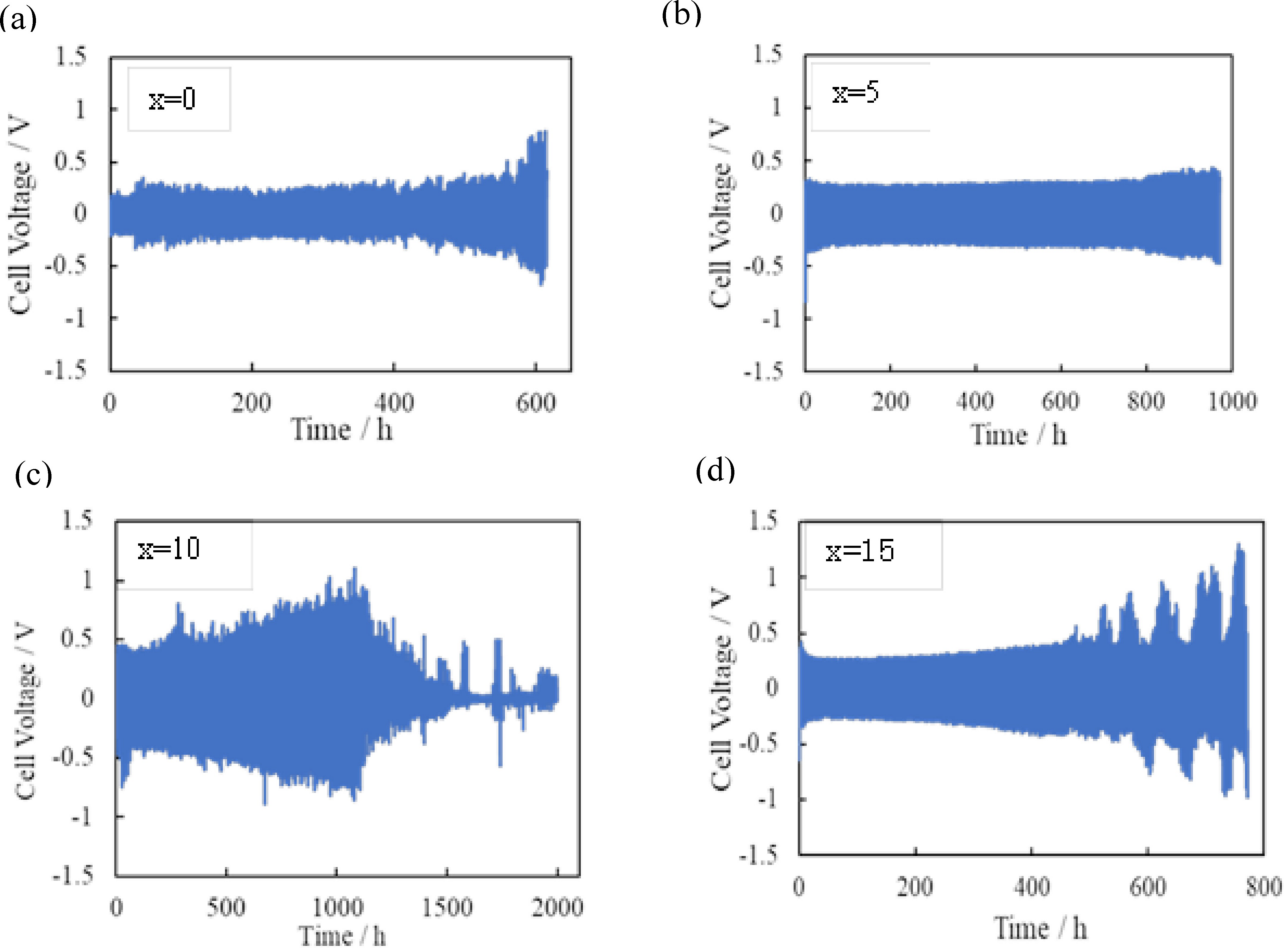
Cycle performance of Li/PEO_18_LiTFSI‐2G4‐10 wt % LLZTO‐x wt % DME at 0.3 mA cm^−2^ for 1 h polarization at 25 °C.

The room temperature lithium electrode performance of the polymer electrolyte is not at acceptable levels.^[29]^ Therefore, the lithium electrode performance of the PEO based electrolytes at a high temperature has been investigated. Only few papers[^25^, ^32^] have reported lithium deposition and stripping cycle performance at a high current density and room temperature for PEO based composite electrolytes with liquid plasticizers. Huo et al.^[32]^ reported the cycle performance of Li/PEO_x_LiTFSI‐15 wt % LLZTO‐19 wt % [EMIM]TF_2_N at 0.5 mA cm^−2^ and 30 °C, where no short‐circuit was observed for 125 h. Zhang et al.^[25]^ also reported that Li/PEO_18_LiClO_4_‐15 wt % LLZTO‐60 wt % SN exhibited steady cycle perfomanc at 0.1 mA cm^−2^ for 1 h polarization and room temperature for 1000 h. In this study, the interface stability of the PEO_18_LiTFSI‐2G4‐10 wt % LLZTO‐x wt % DME electrolyte with a lithium metal electrode was examined by a galvanostatic cycle experiment with a Li/Li symmetric cell at 25 °C. Figure 6 shows the cyclic performance of Li/PEO_18_LiTFSI‐2G4‐10 wt % LLZTO‐x wt % DME at 0.3 mA cm^−2^ for 1 h polarization and 15 min rest at 25 °C. The cell without DME showed a cell potential increase after 550 h polarization, while the cell with 5 wt % DME showed no significant cell voltage increase for around 1000 h polarization. The cell voltage of the cell with 10 wt % DME increeased gradually up to 1000 h and then decresed to around 35 mV after 1500 h cycling. The low cell voltage after 1500 h suggests a short‐circuited. The increease of cell voltage up to 1000 h may be due to decomposition of DME. The composite electrolyte proposed in this study exhibited excellent stability between lithium metal and the composite polymer electrolyte.

**Figure 7:**
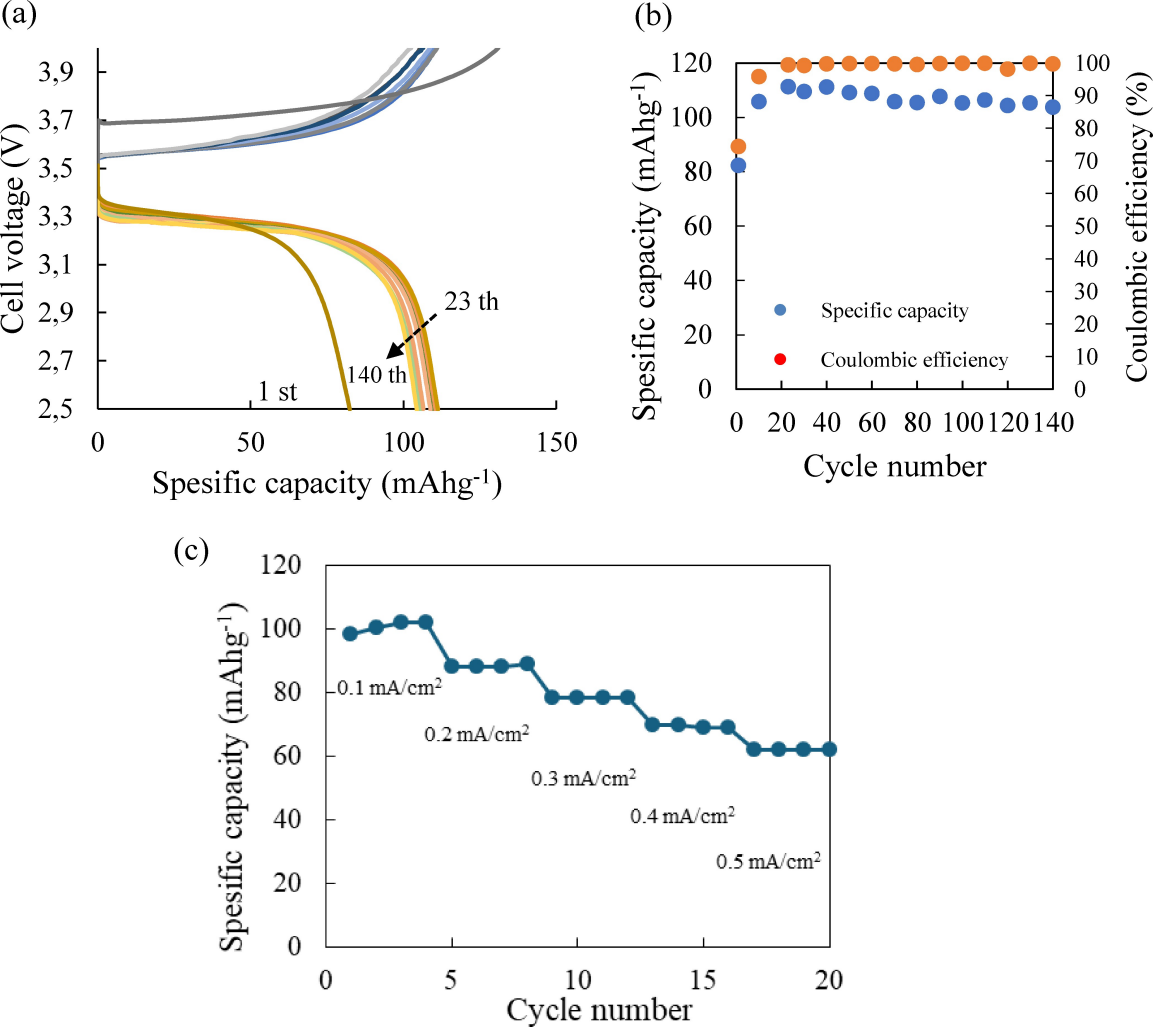
(a) Charge and discharge curves at 0.2 mA cm^−2^, (b) capacity vs. cycle and Coulombic efficiency vs, cycle coucurves and (c) rate performance for a Li/PEO_18_LiTFSI‐2G4‐10 wt % LLZTO‐10 wt % DME/LiFePO_4_ cell.

Figure 7 (a) shows the charge and discharge performance of a Li/PEO_18_LiTFSI‐2G4‐10 wt % LLZTO‐10 wt % DME/LiFePO_4_ cell at 0.2 mA cm^−2^ and 25 °C. The cell could be operated at 0. 2 mA cm^−2^ (~1 C) and room temperture and cycled without degradation for more than 140 cycles. The specific capacity and coulombic efficiency vs. cycle number curves and the rate performance of the cell are shown in Figure 7 (b) and 7 (c), repectivley. After 20 cycles, the steady coulombic efficicecy of 99 % was observed.

## Conclusions

High conductivity flexible polymer composite electrolytes were proposed based on the PEO‐LiTFSI−G4 electrolyte with the addition of LLZTO filler and DME plasticizer. The high conductivity composite polymer electrolyte of PEO_18_LiTFSI‐2G4 was enhanced further by addition of the high lithium‐ion conducting solid electrolyte of LLZTO as a filler material. The electrical conductivity and interface performance between lithium and the composite polymer electrolyte were improved by the addition of the liquid DME plasticizer. The proposed composite polymer electrolyte of PEO_18_LiTFSI‐2G4‐10 wt % LLZTO‐10 wt % DME is flexible with a high electrical conductivity of 6.01×10^−4^ S cm^−4^ at 25 °C. The composite electrolyte showed no short‐circuit for lithium deposition and stripping at 0.3 mA cm^−2^ for 1 h polarization during 1000 h cycling at 25 °C. A full cell with a lithium anode, LiFePO_4_ cathode and the composite electrolyte was successfully operated at 0.2 mA cm^−2^ and room temperature.

## Experimental Section

LLZTO powders were purchased from Toshima Manufacutring Co., Japan. The LLZTO powders were crushed by planetary high‐energy ball milling and the resultant particle sizes were in the range of 0.1 to 5 μm. The SEM images of the powders befor and after ball milling are shown in Figure S5. PEO (Sigma Aldrich, M_v_=6×10^5^ g mol^−1^) and LiTFSI (Wako Chem. Japan) were dried overnight under vacuum at 50 °C and 160 °C, respectively. G4 (Kishida Chemical, Japan) was immersed into activated 0.3 nm molecular sieves for 1 week. Composite polymer electrolytes of PEO_18_LiTFSI‐2G4‐x wt % LLZTO were prepared using a casting method where PEO, LiTFSI, G4, and LLZTO were added to acetonitrile (AN) and stirred for 24 h. The AN solvent was evaporated slowly at room temperature under nitrogen gas flow and then dried at 110 °C for 4 h to remove residual AN. The thickness of the obtained composite polymer electrolytes were around 100 μm. The addition of a small amount of DME was performed by dropping DME onto the PEO_18_LiTFSI‐2G4‐xLLZTO composite electrolyte sheet in an Ar‐filled glove box. The cross‐section SEM images and EDX maps of F and Zr of PEO_18_LiTFSI‐2G4‐10 wt %LLZTO‐10 wt % DME are shown in Figure S6. The EDX maps suggest that LLZTO is homogenosuly distributed over the PEO matrix.

Electrical conductivity measurements of the composite polymer electrolytes were performed using coin‐type cell. The diameter of the electrolyte was 12 cm. The cell impedances were measured using a frequency response analyzer (Solartron 1250) in the frequency range of 0.1 Hz to 1 M Hz and in the temperature range of 25–80 °C. The cell was heated up to 80 °C and then cooled down to room temperature to produce a good contact between the electrode and the composite electrolyte.The conductivity data on the second heating run were recorded in this study. The lithium‐ion transport number t_Li+_ was determined using a Li/PEO_18_LiTFSI‐2G4‐10 wt % LLZTO‐10 wt % DME/Li cell with a combination of AC impedance spectroscopy and the DC polarization method, as proposed by Evans et al.^[30]^ Mechanical tests were performed using a tensile meter to evaluate the tensile strength. X‐ray diffraction (XRD) data were measured using a diffract meater (Bruker DB Advance) with Cu Kα radiation. Cross section of the compsoite electrolyte was investiaged using a Hitachi S 4800 scaning electron microscopy with energy dispersion X‐ray spectrometer (EDX). The lithium plating and striping cycling performance of a symmetrical 2032 type coin cell of Li/PEO_18_LITFSI‐2G4‐10 wt % LLZTO‐x wt % DME/Li was determined at a current density of 0.3 mA cm^−2^ and 25 °C using a potentio‐galvanostat (Biologic VMP X). The The Li/PEO_18_LiTFSI‐2G4‐10 wt % LLZTO‐10 wt % DME/LiFePO_4_ full cell was tested in a 2032 type coin cell. LiFePO_4_ cathode was prepared by mixing LiFePO_4_, carbon, and the composite electrolyte. The weight ratio was 8 : 1 : 1 and 1.30 mg of LiFePO_4_ was charged in the full cell. LiFePO_4_ was purched from Housen, Co. Japan.

## Supporting Information

XRD patterns, Raman spectra, Stress‐strain curves, Conductiviy of PEO_18_LiTFSI‐10 wt.% LLZTO‐xDME, SEM images of LLZTO powder, Cross‐section SEM images and EDX maps of PEO_18_LiTFSI‐2G4‐10 wt % LLZTO‐10 wt % DME.

## Conflict of Interests

The authors declare no conflict of interest

1

## Supporting information

As a service to our authors and readers, this journal provides supporting information supplied by the authors. Such materials are peer reviewed and may be re‐organized for online delivery, but are not copy‐edited or typeset. Technical support issues arising from supporting information (other than missing files) should be addressed to the authors.

Supporting Information

## Data Availability

The data that support the findings of this study are available from the corresponding author upon reasonable request.

## References

[1] D. Castelvecchi , Nature 2021, 596, 336–339. 34404944 10.1038/d41586-021-02222-1

[2] G. Girshkumar , B. McCloskey , A. C. Luntz , S. Swanson , W. Wilcke , J. Phys. Chem. Lett. 2010, 1, 2193–2203.

[3] R. Van Noorden , Nature 2014, 507, 26–28. 24598624 10.1038/507026a

[4] P. G. Bruce , S. A. Freunberger , L. J. Hardwick , J.-M. Tarascon , Nat. Mater. 2012. 11, 19–29. 10.1038/nmat3191 22169914

[5] J. Liu , Z. Bao , Y. Cui , E. J. Dufek , J. B. Goodenough , P. Khalifah , Q. Li , B. Y. Liaw , P. Liu , A. Manthiram , Y. S. Meng , V. R. Subramanian , M. F. Toney , V. V , Viswanathan , M. S. Whittingham , J. Xiao , W. Xu , J. Yang , X.-Q. Yang , J.-G. Zhang , Nat. Energy 2019, 4, 180–186.

[6] Y. Kato , S. Hori , T. Saito , K. Suzuki , m. Hirayama , A. Mitsui , M. Yomenura , H. Iba , R. Kanno , Nat. Energy 2016, 1, 1603–1609.

[7] B. E. Fenton , J. M. Paker , P. V. Wright , Polymer 1973, 14, 589.

[8] M. B. Armand , J. M. Chbagno , M. J. Duclot , Second International Conference on Solid Electrolyte, St. Andrews, 1978, Paper 6.5.

[9] K. Zhaghib , Y. Choquette , A. Guerifi , M. Simoneau , A. Belanger , M. Gutheier , J. Power Sources 1997, 68, 368–371.

[10] X. Yu , A. Manthiram , Energy Storage Mater. 2021, 34, 282–300.

[11] S. Taminato , D. Mori , Y. Takeda , O. Yamamoto , N. Imanishi , ChemistrySelect. 2022, 7, e202201667.

[12] H. Huo , Y. Chen , J. Luo , X. Yang , X. Guo , X. Sun , Adv. Energy Mater. 2019, 9, 1804004.

[13] H. Wang , M. Matsui , Y. Takeda , O. Yamamoto , D. Im , D. Lee , N. Imanishi , Membranes 2013, 3, 298–310. 24957059 10.3390/membranes3040298 PMC4021947

[14] X.-B. Cheng , R. Zhang , C.-Z. Zhao , Q. Zhang , Chem. Rev. 2017, 117, 10403–10473. 28753298 10.1021/acs.chemrev.7b00115

[15] Y. Takeda , O. Yamamoto , N. Imanishi , Electrochem. 2016, 84, 210–218.

[16] H. E. Park , C. H. Hong , W. Y. Yoon , J. Power Sources 2008, 178, 765–768.

[17] S. Choudury , L. A. Archer , Adv. Electron. Mater. 2016, 2, 1500246.

[18] J. Qian , W. A. Hendarson , W. Xu , P. Bhattacharys , M. Engelhard , O. Borodin , J. G. Zhang , Nat. Commun. 2015, 6, 6362. 25698340 10.1038/ncomms7362 PMC4346622

[19] S. Lin , H. Wang , N. Imanishi , T. Zhang , A. Hirano , Y. Takeda , O. Yamoto , J. Yang , J. Power Sources 2011, 196, 7681–7686.

[20] Y. Bae , J. Li , X. Zhang , F. Zhao , Y. Shi , J. B. Goodenough , Angew. Chem. Int. Ed. 2018, 57, 2096–2100. 10.1002/anie.201710841 29314472

[21] X. Song , T. H. Zhang , R. Z. Fan , J. Biao , S. H. Huang , J. Travas-Sejdie , W. Gao , P. Cao , Solid State Ionics 2023, 403, 116410.

[22] S. Li , S.-Q. Zhang , L. Shen , Q. Liu , J.-B. Mo , W. Lv , Y.-B. He , Q.-H. Yang , Adv. Sci. 2020, 7, 1903088. 10.1002/advs.201903088 PMC7055568 32154083

[23] L. Chen , Y. Li , S.-P. Li , L.-Z. Fan , C.-W. Nan , J. B. Goodenough , Nano Energy 2018, 46, 178–184.

[24] V. T. Luu , Q. H. Nguyen , M. G. Park , H. L. Nguyen , M.-H. Seo , S.-K. Jeong , N. Cho , Y. W. Lee , Y. Cho , S. N. Lim , Y.-S. Jun , W. Ahn , J. Mater. Res. Tech. 2021, 15, 5849–5863.

[25] X. Zhang , C. Fu , S. Cheng , C. Zhang , L. Zhang , M. Jiang , K. Wang , Y. Ma , P. Zuo , C. Du , Y. Gao , G. Yin , H. Huo , Energy Storage Mater. 2023, 56, 121–131.

[26] S. Liu , W. Liu , D. Ba , Y. Zhao , Y. Ye , Y. Li , J. Liu , Adv. Mater. 2023, 35, 2110423. 10.1002/adma.202110423 35949194

[27] S. Li , S.-Q. Zhang , L. Shen , Q. Liu , J.-B. Ma , W. Lv , Y.-B. He , Q.-H. Yang , Adv. Sci. 2020, 7, 1903088. 10.1002/advs.201903088 PMC7055568 32154083

[28] H. Huo , Y. Chen , J. Luo , X. Yang , X. Guo , X. Sun , Adv. Energy Mater. 2019, 9, 1804004.

[29] R. Chen , Q. Li , X. Yu , L. Chen , H. Li , Chem. Rev. 2020, 120, 6820–6877. 31763824 10.1021/acs.chemrev.9b00268

[30] J. Evans , C. A. Vincent , P. G. Bruce , Polymer 1987, 28, 2324–2328.

[31] T. Zhang , N. Imanishi , A. Hirano , Y. Takeda , O. Yamamoto , Electrochem. Solid-State Lett. 2011, 14, A45–A48.

[32] H. Huo , N. Zhao , J. Sun , F. Du , Y. Li , X. Guo , J. Power Sources 2017, 372, 1–7.

[33] K. Z. Walle , L. M. Babula , S.-H. Wu , W.-C. Chen , R. Jose , S. J. Lue , J.-K. Chang , C.-C. Yang , ACS Appl. Mater. Interfaces 2021, 12, 2507–2520. 10.1021/acsami.0c17422 33406841

