# Supplementary material for: Flexible High Lithium‐Ion Conducting PEO‐Based Solid Polymer Electrolyte with Liquid Plasticizers for High Performance Solid‐State Lithium Batteries
Source: ChemistryOpen. 2024 Apr 15;13(9):e202400041. doi: 10.1002/open.202400041 (PMC11633345; doi:10.1002/open.202400041)
Supplement: Supplementary file 1 — Supporting Information [file OPEN-13-e202400041-s001.pdf]

ChemistryOpen

Supporting Information

Flexible High Lithium-Ion Conducting PEO-Based Solid Polymer Electrolyte with Liquid Plasticizers for High Performance Solid-State Lithium Batteries

Ayaka Abe, Daisuke Mori,* Zhichao Wang, Sou Taminato, Yasuo Takeda, Osamu Yamamoto,* and Nobuyuki Imanishi

Chemstry Open

Suporting information

Flexible high lithium-ion conducting PEO-based solid polymer electrolyte with garnet-type lithium-ion conducting solid electrolyte and liquid plasticizers for high performance solid-state lithium batteries

Ayaka Abe[a], Daisuke Mori*[a],Zhicho Wang, Sou Taminato[a], Yasuo Takeda[a], Osamu Yamamoto*[a], and Nobuyuki Imanishi[a][b]

[a] A. Abe, Prof. Dr. D. Mori, Z. Wang, Prof. Dr. S. Taminato, Pro. Dr. Y. Takeda, Prof. Dr. O. Yamamoto, Prof. Dr. N. Imanishi
Graduate School of Engineering, Mie University, Tsu, Mie, 514-8507, Japan
[b] Prof. Dr. N. Imanishi
Research Center for Integrated Materials and Interfaces for Sustainable Energy, Mie University, Tsu, Mie, 514-8507, Japan
E-mail: daisuke.mori@chem.mie-u.ac.jp,yamamoto@chem.mie-u.ac.jp

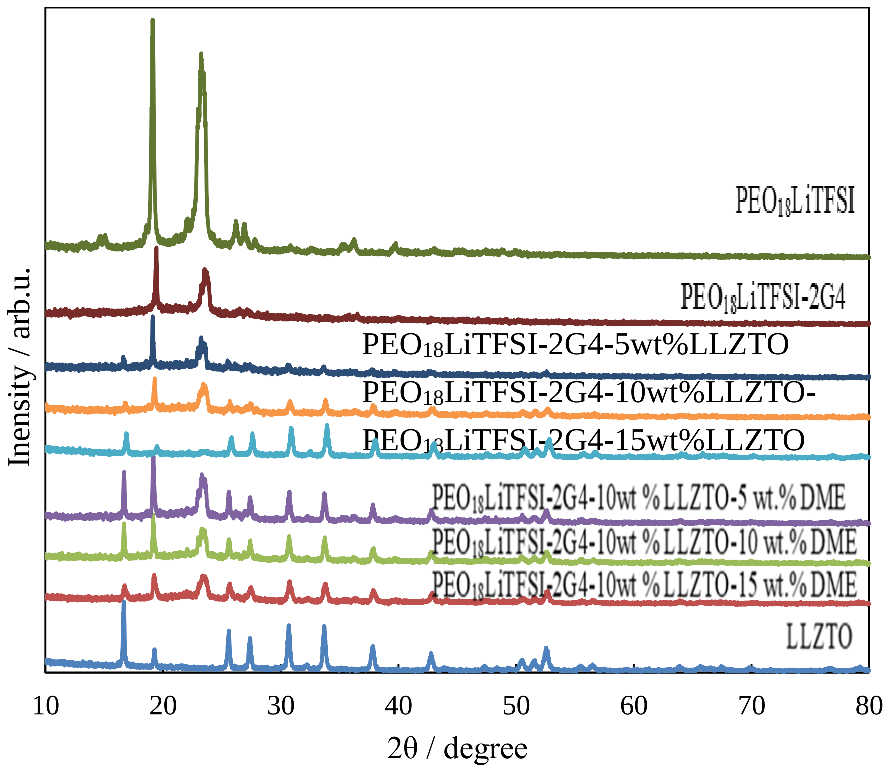

Figure S1. XRD patterns for $PEO_{18}LiTFSI$-2G4 and $PEO_{18}LiTFSI$-2G4-10 wt% LLZTO, and $PEO_{18}LiTFSI$-2G4-10 wt%LLZTO-10 wt% DME.

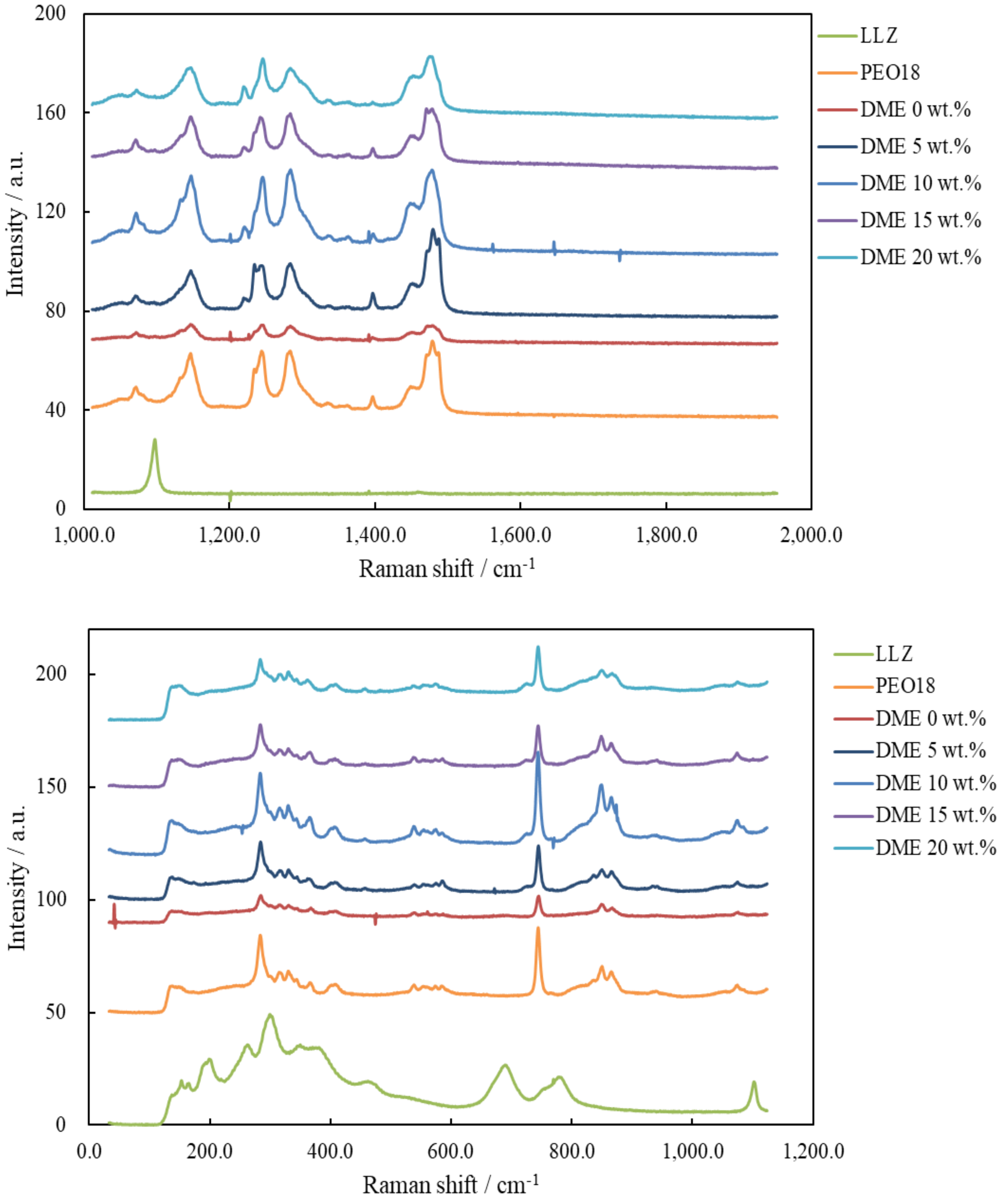

Figure S2. Raman spectra for $PEO_{18}LiTFSI$, LLZTO, $PEO_{18}TFSI$-2G4-10 wt% LLZTO-xDME at room temperature.

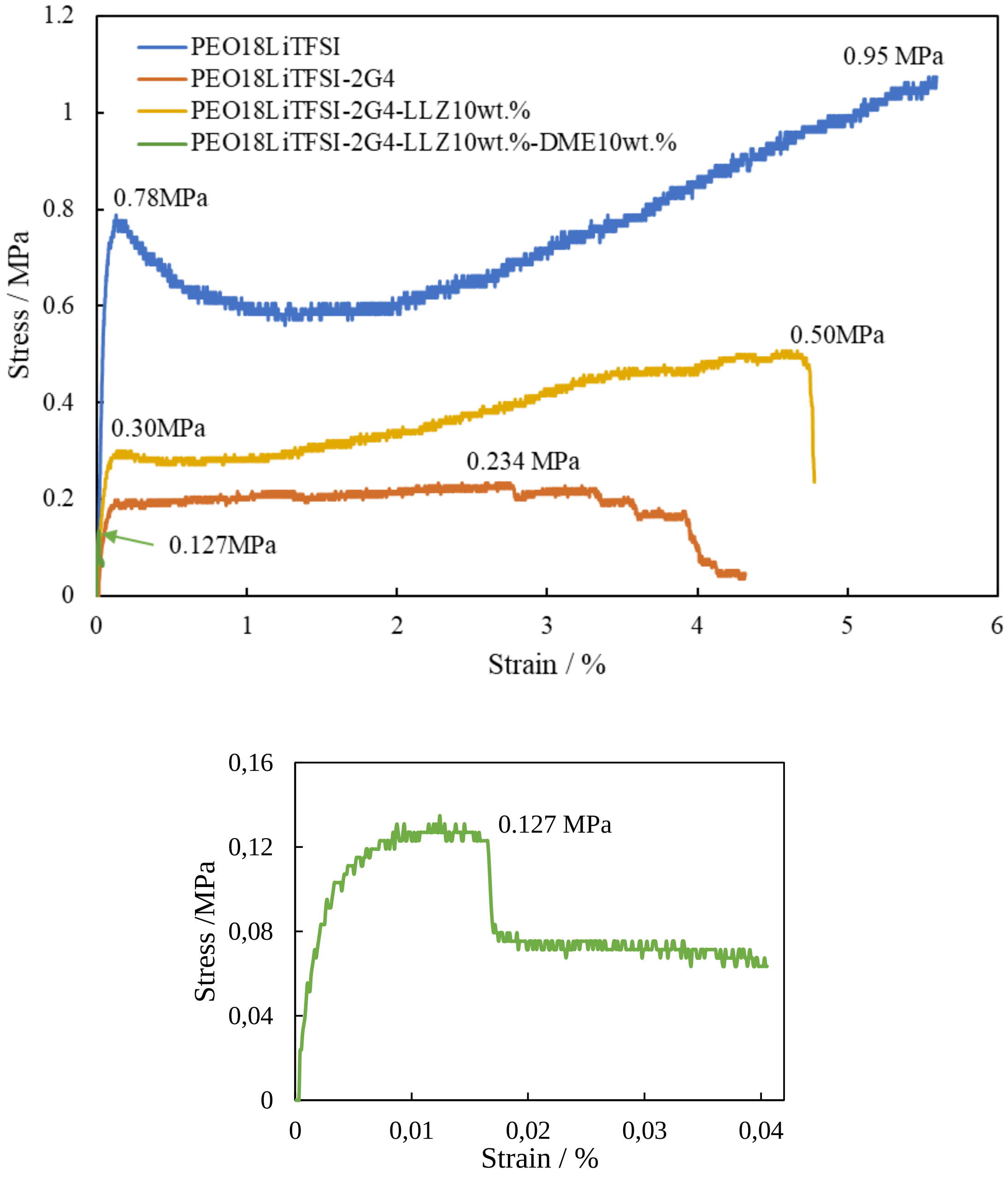

Figure S3. Stress-strain curves for $PEO_{18}LiTFSI$. $PEO_{18}LiTFSI$-2G, $PEO_{18}LiTFSI$-2G4-10 wt% LLZTO, and $PEO_{18}LiTFSI$-2G4-10 wt% LLZTO-10 wt% DME.

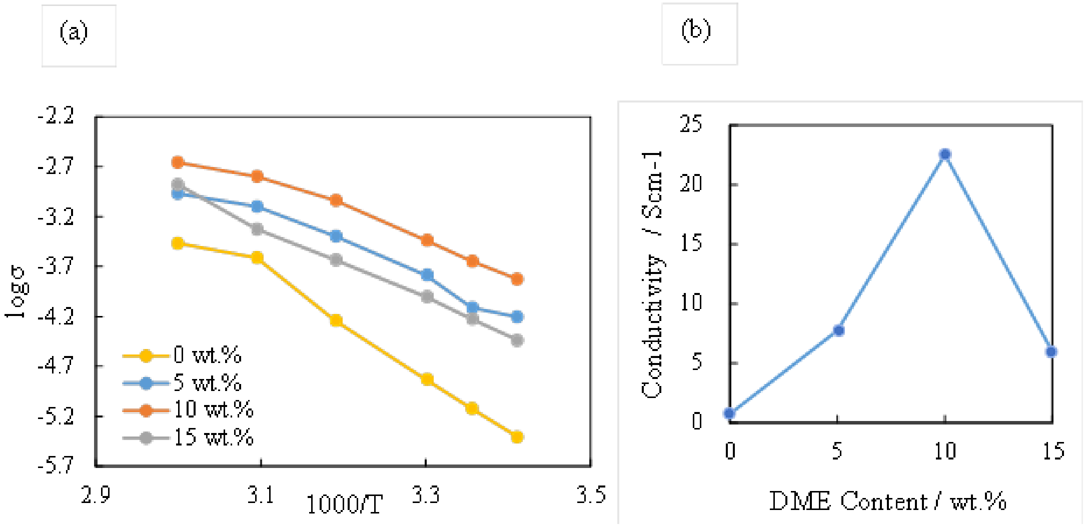

Figure S4 (a) Temperatur dependence of electrcial conductivity and (b) conpositional depencdece of the electrical condcutivity at 25 °C for $PEO_{18}LiTFSI$-10wt % LLZTO-xDME

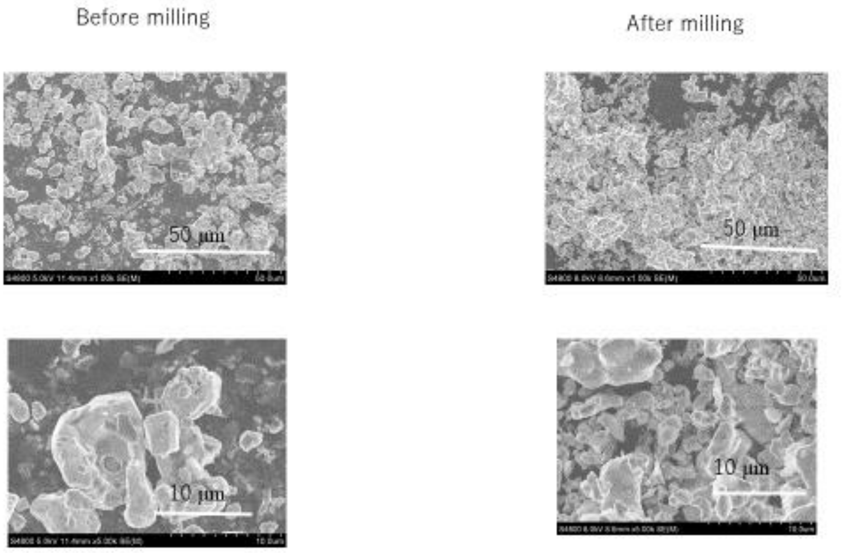

Figure S5 SEM images of LLZTO powders before and after milling using a planetary high-energy ball milling

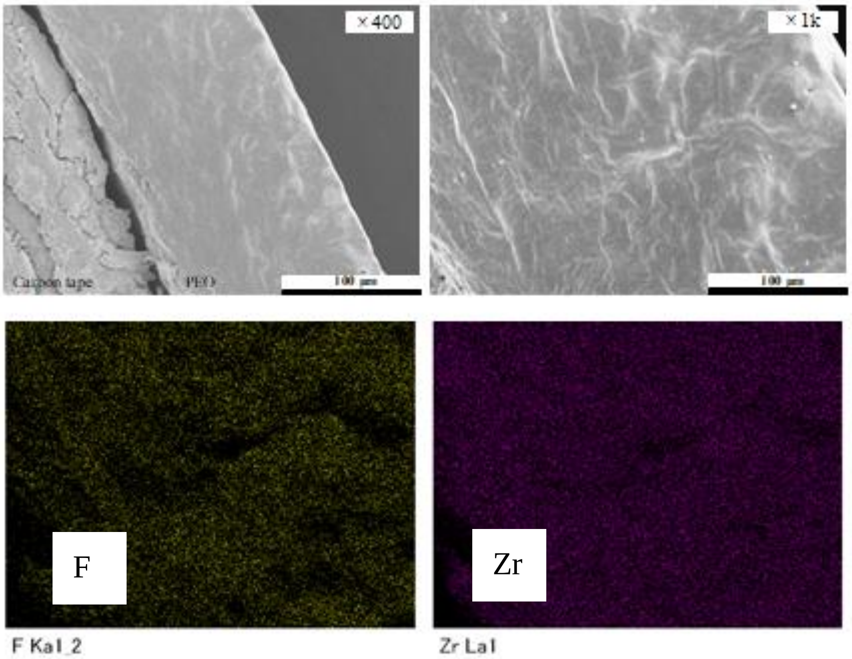

Figure S6　Cross-section SEM images and EDX maps Fe and Zr of PEO_{18}LiTFSI-2G4-10 wt.% LLZTO-10 wt.% DME
